# Tone Decay Reconsidered: Preliminary Results of a Prospective Study in Hearing-Aid Users with Moderate to Severe Hearing Loss

**DOI:** 10.3390/jcm13020500

**Published:** 2024-01-16

**Authors:** Florian Herrmann Schmidt, Thomas Hocke, Lichun Zhang, Wilma Großmann, Robert Mlynski

**Affiliations:** 1 Department of Otorhinolaryngology, Head and Neck Surgery, ‘Otto Körner’, Rostock University Medical Center, Doberaner Strasse 137-139, 18057 Rostock, Germany; lichun.zhang@med.uni-rostock.de (L.Z.); wilma.grossmann@med.uni-rostock.de (W.G.); robert.mlynski@med.uni-rostock.de (R.M.); 2 Cochlear Deutschland GmbH & Co. KG, Karl-Wiechert-Allee 76, 30625 Hannover, Germany; thocke@cochlear.com

**Keywords:** tone decay, suprathreshold diagnostics, retrocochlear disorders, cochlear implant, hearing aid

## Abstract

Among hearing aid (HA) users, there is a considerable variability in word recognition scores (WRSs). This variability is most pronounced among individuals with moderately severe to severe hearing loss. The variability cannot be adequately explained by factors such as pure-tone audiogram, audiogram type or age. This prospective study was designed to investigate the relationship between tone decay (TD) and WRS in a group of HA users with corresponding pure-tone hearing loss. The study population included 22 patients with hearing loss between 50 and 80 dB HL. Aided WRS, unaided WRS and TD were assessed for both ears. TD was found to be frequency-dependent. TD and WRS were correlated, with up to R = −0.66. The TD test was revealed to be a feasible method for explaining variability in WRS among HA users with hearing loss below 80 dB. This may contribute to improved differential diagnostics. The TD test may thus offer a better understanding of the limitations of HA use in the context of cochlear implant candidacy assessment for HA users.

## 1. Introduction

Addressing hearing loss and effectively managing it with hearing aids is vital for improving the quality of life of individuals with hearing impairments. This is especially crucial in cases of severe hearing loss, where the impact on speech comprehension is significantly heightened. A particularly interesting aspect within this field is the variation in speech comprehension among a specific subgroup of individuals characterised by a pure-tone average for thresholds at 0.5, 1, 2 and 4 kHz (4FPTA) ranging from 60 to 80 dB HL, as recent studies have shown [1,2]. It is important to note that this variability in speech comprehension persists even in the presence of adequate hearing aid (HA) intervention. This variability has been found with respect to two measures, namely (i) the 4FPTA and (ii) the difference between the maximum word recognition score, WRS_max_, and the aided score, WRS_65_(HA), at a conversational level [1,3,4,5]. The first concepts to address the discrepancy between WRS and pure-tone thresholds were introduced by Carhart [6]: for word recognition in quiet, this was referred to as “loss of acuity”, and a second component caused by impaired processing of audible speech signals was referred to as “loss of clarity”. Plomp [7] named these components of hearing loss “attenuation” (class A) and “distortion” (class D). Attenuation can be quantified by pure-tone audiometry. The distortion component characterises the negative impact of reduced temporal and frequency resolution. Furthermore, Plomp [7] stated that the distortion component has a detrimental effect on WRS in quiet as well. Consequently, the distortion component explains the deterioration in speech comprehension that is not expressed by attenuation (4FPTA).

The term “distortion” can be applied to denote individuals who exhibit deficient speech comprehension within the above-mentioned subgroup (persons with 60–80 dB HL 4FPTA). These deficits are thought to have their root causes in disturbed temporal processing and limited frequency resolution of the auditory periphery. A plausible explanatory framework for this phenomenon involves the notion of perceptual decay. In such instances, the perception of loudness diminishes over a brief period, despite consistent sound-level presentation. The tone decay test (TDT), introduced by Carhart [8], entails the presentation of a continuous tone at 5 dB SL (sensation level), with successive increments in intensity until a stable perceptual threshold is achieved. The cumulative increase in intensity resulting from this procedure is termed tone decay (TD).

Abnormal TD may be inferred when attenuation exceeds the threshold of 15 dB. Huss et al. [9] found that abnormal TD becomes more prevalent when hearing loss exceeds 50 dB HL. Importantly, TD is not manifested uniformly across all frequencies and intensities; rather, it is particularly pronounced at higher frequencies compared with lower ones. A decline in loudness perception, known as loudness adaptation, can also occur at higher sound levels, although with less prominence in comparison with stimuli near the perceptual threshold.

The underlying physiological cause of TD has yet to be definitively elucidated. However, the initial observation that low levels and high frequencies are more susceptible to TD led to the formulation of the “restricted pattern” hypothesis [10,11]. This hypothesis suggests that for tones with minimal sensation levels or frequencies that stimulate the basal end of the cochlea, the spatial excitation pattern on the basilar membrane evoked by continuous tones is highly confined. Nonetheless, Wynne et al. [12] posit that TD can be attributed either to direct damage to inner hair cells or to the auditory nerve. Their findings suggest that TD at low frequencies arises from disruptions in ribbon synapses, while high-frequency TD is more likely to be associated with auditory nerve disruptions. Nevertheless, a consensus seems to have formed in that either inner hair cells or retrocochlear structures are involved in the TD phenomenon.

As TD increases, a corresponding decline in both unaided and aided speech recognition can be expected. However, it remains unclear to what extent this decrease can be attributed solely to perceptual decay. Furthermore, it is unclear whether this effect persists even after the provision of an HA and, if so, which frequency range is particularly susceptible to heightened challenges in speech comprehension.

To our knowledge, up to now, there has been no investigation of TD in order to explain the variability in WRS_max_ and WRS_65_(HA) beyond the impact of 4FPTA. This study was therefore undertaken to investigate a patient cohort characterised by hearing loss (4FPTA) ranging from 50 to 80 dB HL and, after the completion of measurements and discharge of patients, to compare TD with unaided and aided word recognition scores.

## 2. Materials and Methods

### 2.1. Patient Characteristics

Twenty-two patients were included in this prospective study: ten males and twelve females. Their mean age was 67.6 ± 14.5 years, ranging from 37 to 88 years. Five subjects had both ears included in this study, resulting in a total of 12 left ears and 15 right ears. The patients visited the clinic for one of two reasons: they were recruited in one of the local hearing aid shops or they visited our clinic for HA evaluation between November 2021 and June 2023. A hearing care professional ensured that the hearing aids were properly fitted for all participants. A technical fitting with an ear mould suitable for the individual’s hearing loss was conducted to minimise any impact of processor fitting on performance. The single exclusion criterion was single-sided deafness according to the definition given by Arndt et al. [13].

### 2.2. Audiological Parameters

All participants underwent audiometric tests in both ears using a clinically calibrated audiometer (AT900, Auritec GmbH, Hamburg, Germany). Pure tones were presented at frequencies of 0.125, 0.25, 0.5, 0.75, 1, 2, 3, 4, 6 and 8 kHz through a headphone (DT48; Beyer, Heilbronn, Germany). To prevent the perception of pure tones through bone conduction in the contralateral ear, masking was initiated when there was a difference of 40 dB between the ipsilateral hearing threshold and the contralateral bone conduction threshold. Subsequently, speech recognition was evaluated by measuring the word recognition score (WRS). In the unaided condition, different sound levels were presented, also through the headphone, to find the highest word recognition score (WRS_max_). The participant′s discomfort threshold served as the upper limit for the levels. A minimum level of 95 dB SPL was presented to all participants. The aided word recognition score, WRS_65_(HA), was measured in quiet unilaterally at a presentation level of 65 dB sound pressure level (SPL) in free field. The contralateral ear was masked appropriately. The speech test signal (Freiburg Monosyllable Test) was presented frontally in a soundproof room (5 × 6 × 2.5 m).

### 2.3. Tone Decay Test

For the tone decay test (TDT) [8], an AT900 audiometer was used according to routine procedures, as follows. Before TD testing, an audiogram is obtained. The patient is then familiarised with the TDT measurement procedure: “You will hear a very soft sound. Press the button as soon as the sound is no longer audible” [14].

The TDT is then performed for the frequencies 1, 1.5, 2, 3 and 4 kHz, if possible. This depends on the corresponding threshold with respect to the audiometer limits of 110 dB HL. For the AT900 we used, this procedure is integrated according to the recommendations of Lehnhardt [15]: At each frequency, the starting level is set at 5 dB SL (dB sensation level). The test begins with the presentation of a continuous tone at the starting level for a maximum of 60 s. The patient confirms briefly that this level is audible. When the patient presses the button to indicate that the tone is no longer audible within 60 s, the procedure is repeated. For this, a new starting level increased by 5 dB is presented. The test ends when the patient continues to report audibility for at least 60 s, or when the maximum permissible volume of 110 dB has been reached. The difference between the starting level and the resulting level at the end of this procedure is referenced as TD.

**Figure 1:**
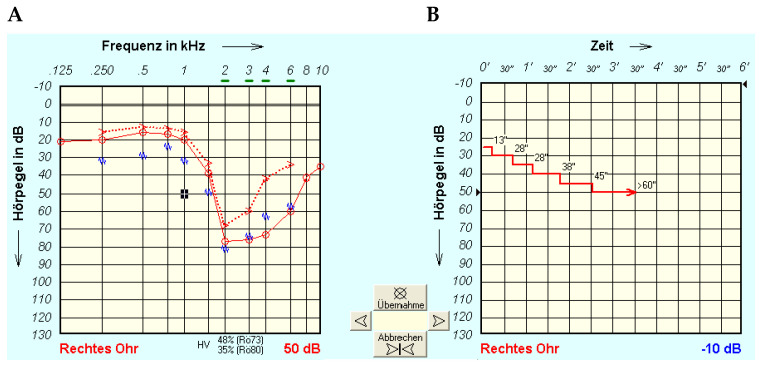
Representative tone decay test performed at 1 kHz and final stimulation at 50 dB HL, 30 dB above hearing threshold. (**A**) Hearing threshold (red circle), the bone conduction threshold (red arrow pointing to the right), and the contralateral masking level (blue wave). (**B**) Change if sound level over time during the tone decay test (TDT).

Figure 1 shows an example of measurement in a study participant (right ear) with a very strong tone decay. The left-hand panel (A) shows pure-tone hearing loss (air and bone conduction together with the contralateral masking noise). The right-hand panel (B) shows tone decay, here for 1 kHz, over time. In this example, the last presentation level heard for at least 60 s was 50 dB, and TD at 1 kHz was 25 dB.

The performance of the test is limited by the degree of hearing loss due to the 110 dB level limitation and, in the case of asymmetrical hearing loss, by the side difference of the threshold as determined by audiometry conducted before the test (>40 dB was considered excessive). This is because a test involving active masking of the contralateral ear is likely to be less reliable. In addition, with severe hearing loss, auditory fatigue is not always maximally detectable due to the level limitation. This sealing effect must be taken into account when interpreting the results. If tinnitus is present, the test cannot be performed for the frequencies affected.

### 2.4. Possible Characteristics of Tone Decay Measurements

**Figure 2:**
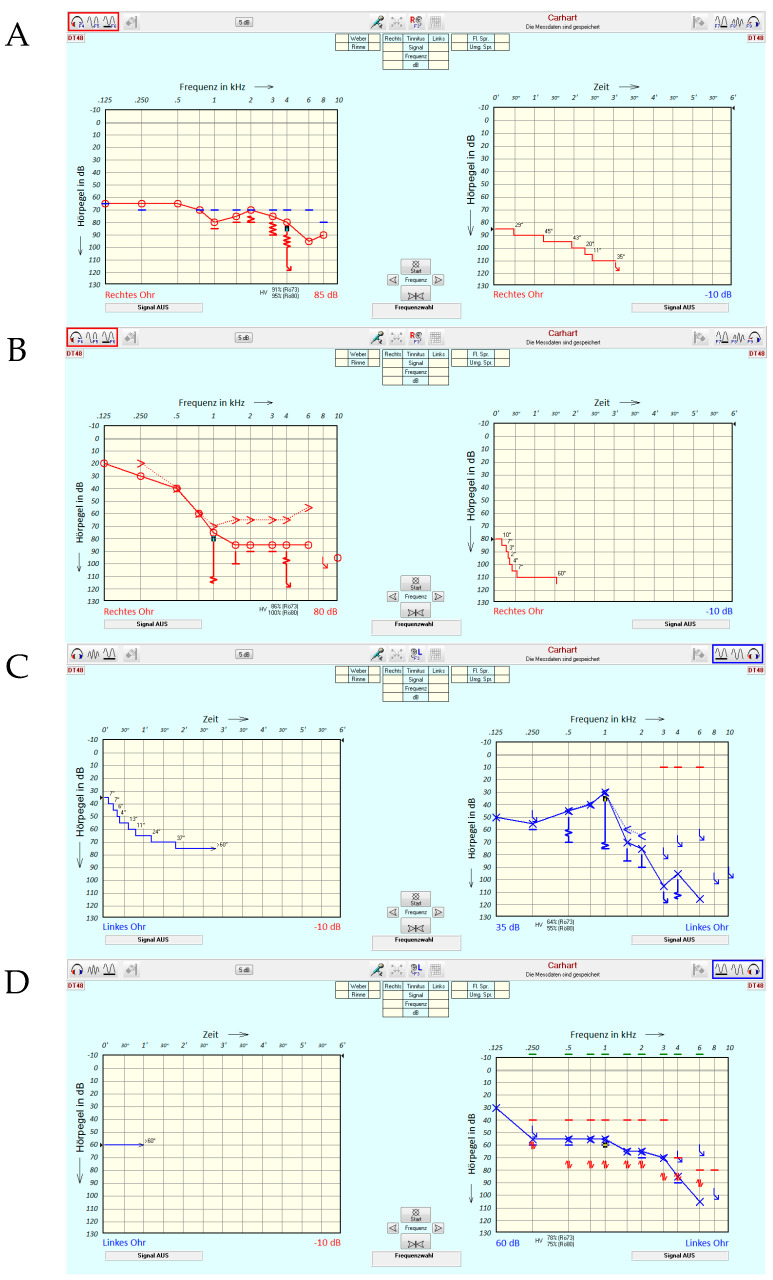
Case examples of different tone decay test (TDT) characteristics. (**A**–**D**) Case examples. For right-ear cases (red), the graph on the left shows the pure-tone audiogram and that on the right shows the time course of the TDTs. The ordinate corresponds to the presentation level in dB HL. The colours red and blue correspond to measurements of the right and left ear, respectively. The actual test frequency is indicated by a black square symbol on the left while the precise course of TDT is displayed on the right for right ears and vice versa for left ears. The highlighted squares in red and blue in the upper right and left corner visualize the measurement condition.

Figure 2 illustrates a selection of possible characteristics of TD measurements. It underlines the degree to which all the information contained in the TDT is reduced in this first feasibility study, where only the final extent of TD [dB] is evaluated.

In case example A, a patient (right ear) with pantonal hearing loss of 4FPTA = 74 dB is presented. TD, here assessed at 4 kHz as indicated by the small black square on the left, is apparently larger than the audiometer limits allowed for measurement. In this case, the TDT yields 25 dB, thereby underestimating the impact of disturbed processing on tone perception. The test time for 4 kHz can be seen in the right-hand part of the figure and was more than 3 min. Case example B shows a patient (right ear) with a steep sloping audiogram and a TD of 30 dB. A special characteristic of this case is the rapid speed of threshold deterioration, as indicated in the right-hand part of Figure 2B. Sometimes, the test tone of 1 kHz was audible more or less for 2 s. Case example C (left ear) shows that this kind of rapid decline is not necessarily connected to a threshold of 75 dB, as in case example B, but may already be apparent at a threshold of 30 dB. As shown in Figure 2C, the test tone was heard for longer with increasing presentation level. However, an audibility of at least 60 s was only reached at 30 dB SL. Finally, case example D shows a patient (left ear) with a moderate sloping audiogram without any TD.

## 3. Results

The demographic and audiometric data of the study patients are displayed in Table 1.

The clinical characteristics of the patients did not exhibit any correlation with WRSs. We examined factors such as age, sex, and ear side, comparing them with both WRS_max_ and WRS_65_(HA). No significant correlation was identified between age and either WRS_max_ or WRS_65_(HA). Similarly, a two-sided *t*-test for ear and sex characteristics revealed no significant differences.

### 3.1. Pure-Tone and Speech Audiometry

**Figure 3:**
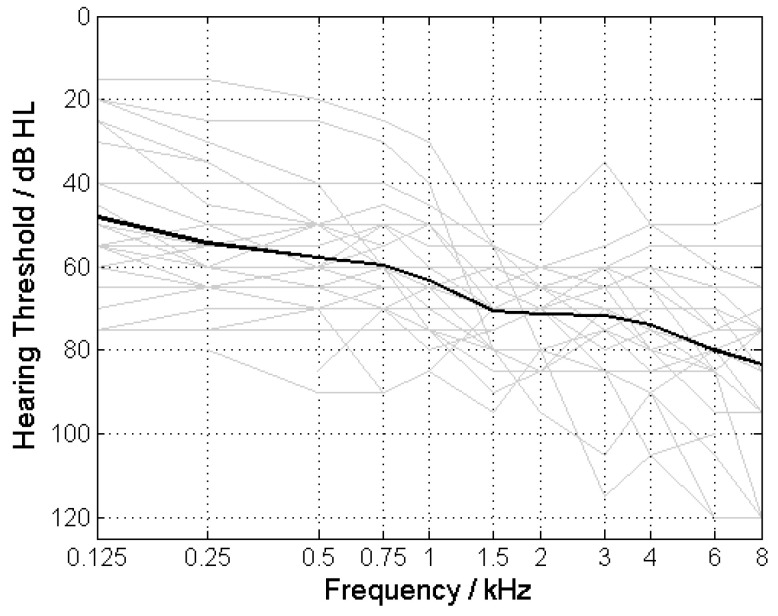
Pure-tone thresholds of the study participants.

**Figure 4:**
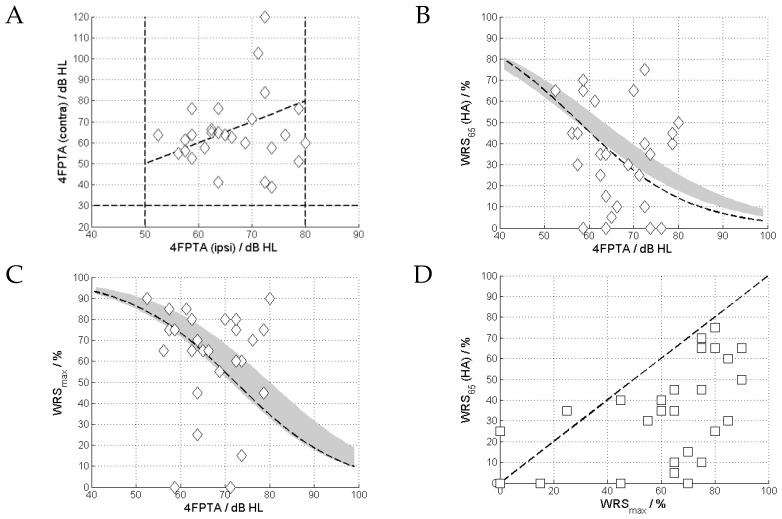
Relationship between four-frequency pure-tone average (4FPTA) and the two word recognition scores. (**A**): Ipsi- and contralateral 4FPTA. (**B**): The aided score, WRS_65_(HA), plotted against 4FPTA; the grey area shows the average WRS_max_(HA) as a function of 4FPTA with the corresponding 95% confidence interval [16], and the hatched line shows the logistic regression of the data of this study. (**C**): The maximum word recognition score, WRS_max_, plotted against 4FPTA; grey area and hatched line as in upper right. The solid line shows the logistic regression of the data of this study. (**D**): Relationship between WRS_65_(HA) and WRS_max_.

All ears had moderate to severe hearing loss (Figure 3) and a 4FPTA between 50 and 80 dB HL, with an average 4FPTA of 66.6 ± 7.7 dB HL (mean ± standard deviation). A 4FPTA of at least 30 dB HL was present in the contralateral ear, with an average of 65 ± 17.2 dB HL (Figure 4A). The word recognition score at 65 dB with HA (WRS_65_(HA)) was 34.1% ± 23.5% (Figure 4B), whereas the maximum word recognition score (WRS_max_) was 61.9% ± 25.2% (Figure 4C). In almost all cases, WRS_65_(HA) was below WRS_max_ (Figure 4D).

Figure 3 shows the pure-tone thresholds for the ears included in this study. According to the inclusion criteria, the 4FPTA ranged from 50 to 80 dB.

Figure 4A shows the relationship between the 4FPTAs of the ipsilateral (included case) and contralateral ears. The vast majority of cases show asymmetric 4FPTAs of up to 70 dB. Figure 4B shows WRS_65_(HA) vs. 4FPTA. The majority (24) of cases exhibit a WRS_65_(HA) below WRS_max_. Despite the narrow 4PTFA inclusion band (50 to 80 dB HL), we see a highly variable WRS_max_, from 0 to 90%, with a variability of the aided scores from 0 to 75% (Figure 4C). Figure 4D shows the relationship between WRS_65_(HA) and WRS_max_. For the majority of cases (24/27) WRS_65_(HA) did not reach WRS_max_.

### 3.2. Tone Decay

**Figure 5:**
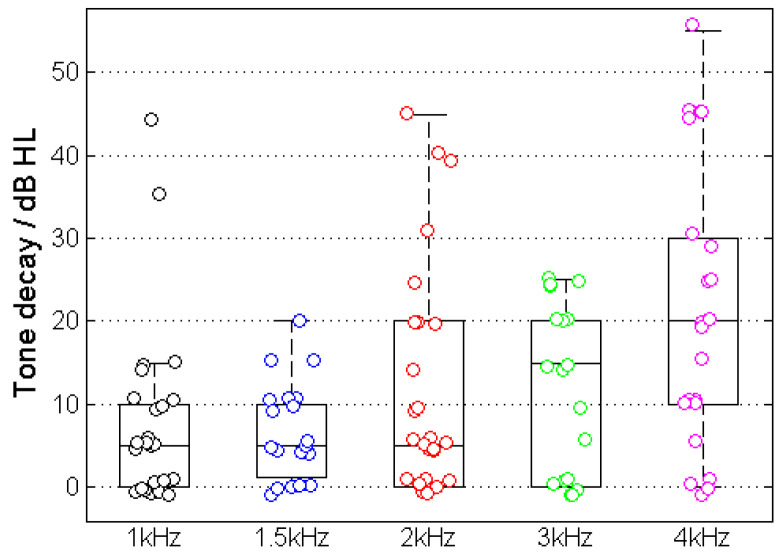
Tone decay at different test frequencies.

Figure 5 shows the results of TD measurement for the example in Figure 1. The majority of cases showed a TD with higher incidence and amplitude at higher frequencies. The measured TD increased at higher frequencies and resulted in TD_1kHz_ = 8.0 ± 10.6 dB, TD_1.5kHz_ = 6.8 ± 5.8 dB, TD_2kHz_ = 12.4 ± 14.0 dB, TD_3kHz_ = 12.2 ± 10.3 dB and TD_4kHz_ = 21.1 ± 17.2 dB.

**Figure 6:**
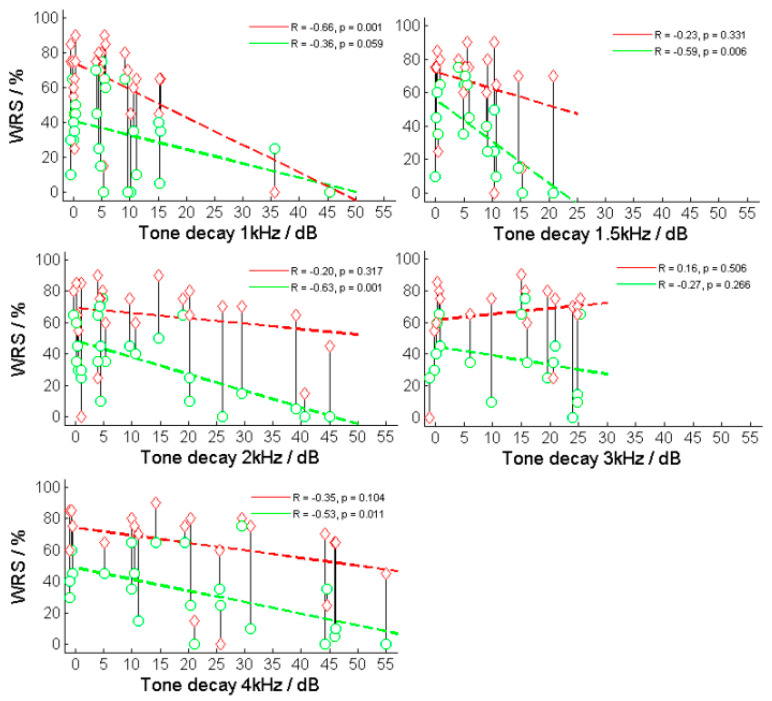
Relation between maximum and aided word recognition scores (WRS_max_ and WRS_65_(HA)) and tone decay (TD) at different frequencies. The red diamonds and lines correspond to the relationship between TD and WRS_max_, while the green circles and lines correspond to the relationship between TD and WRS_65_(HA).

The correlations between the TDs for each frequency and speech recognition for both WRS_65_(HA) and WRS_max_ were investigated. Significant correlations were found for WRS_65_(HA) and TD_1.5kHz_ (R = −0.60, *p* < 0.01) and TD_2kHz_ (R = −0.63, *p* < 0.001) (Figure 6) as well as WRS_max_ and TD_1kHz_ (R = −0.66, *p* < 0.001). The same result was obtained from the frequency-specific comparison after the subjects had been grouped into normal and abnormal TD, resulting in WRS_65_(HA) and TD_1.5kHz_ (*p* = 0.005) and TD_2kHz_ (*p* = 0.006) as well as WRS_max_ and TD_1kHz_ (*p* = 0.006).

Figure 5 shows the relationship between the two WRSs (WRS_max_ and WRS_65_(HA)) and TD for various test frequencies.

## 4. Discussion

### 4.1. Tone Decay and Speech Comprehension

In this study, we explored the relationship between speech comprehension in individuals with moderately severe to severe hearing loss (4FPTA = 50–80 dB HL) and tone decay within the frequency range 1–4 kHz. Our hypothesis posits that a portion of the considerable variability in speech comprehension within this threshold range for hearing loss could be elucidated by factors such as tone decay (TD). The large variability (90 percentage points for WRS_max_ and 75 percentage points for WRS_65_(HA)) in the inclusion range for this study (50–80 dB HL) corresponds to previously reported WRSs in a larger population of HA users [1,3,4,5,17,18]. This applies also to the difference between maximum and aided WRSs. We conclude that the results of our study are applicable to the population of hearing aid users typical for an ENT department of a maximum-care hospital with a cochlear implant programme.

The key finding of this study is the negative correlation between TD and speech comprehension. A consistent trend is evident across all frequencies, with the 1–2 kHz range demonstrating the most clearly significant correlation. This finding aligns with expectations, considering the critical importance of this frequency range for speech comprehension. Moreover, our investigation may indicate that the impact of TD on speech comprehension is more pronounced when using HAs compared with situations without them in the frequency range between 1.5 and 3 kHz. Notably, during the unaided speech comprehension test, we examined WRS_max_ at levels typically reaching the discomfort level, whereas in the aided test, speech was assessed at 65 dB SPL in free field. In cases with unstable thresholds, which correspond to higher tone decays, the fitting of the HA to the threshold becomes imprecise, resulting in inaccurate assumptions for frequency-specific amplification in the presence of TD. Additionally, TD occurs more rapidly with active HAs, as they consistently stimulate close to and beyond the threshold. In contrast, WRS_max_ appears more resilient against TD, possibly owing to the consideration of several levels within the measurement paradigm.

This outcome highlights the constraints of traditional approaches to HA programming when confronted with TD, shedding light on why this particular patient group may no longer be deemed suitable for HA fitting and might be more inclined toward consideration for cochlear implant (CI) fitting. Nevertheless, exploring alternative fitting strategies that take TD into account is a plausible avenue. Such strategies would require the recalibration of thresholds and corresponding amplification, tailored to the frequency-specific challenges posed by TD. To achieve this, the frequency- and level-specific temporal trajectory of TD would need to be recorded individually, coupled with an understanding of possible threshold recovery processes.

### 4.2. Application of Tone Decay Assessment in a Changing Patient Population

One important aspect of HA evaluation in our clinic is the assessment of cochlear implant (CI) candidacy. The most recent German CI guidelines [19] support CI implantation up to WRS_65_(HA) ≤ 60% regardless of pure-tone thresholds. In the past decade, this has led to a growing number of CI candidates among cases with considerable aided WRS [20,21,22,23]. Together with a higher preoperative WRS_65_(HA), this also extends CI provisions to some patients with a 4FPTA of 50 dB HL [20,21,22,23]. These patient characteristics would allow for improved preoperative differential diagnoses in CI candidates with good 4FPTA and poor WRS. Since some of these diagnostic tests for CIs (of which TDT is one example) require a sufficient amount of residual hearing, TDT may offer a chance—and may meet the clinical need—to explain the variability in WRS_65_(HA). If, for instance, a CI candidate shows a PTA and disproportional loss in aided speech recognition, a TDT should be applied. A considerable TD may limit the residual dynamic of the patient’s HA or alternative hearing system to such a degree that a CI can be considered a better alternative [16,24,25,26]. Potentially, the decision to conduct another HA trial can be based on such differential diagnostics.

Additionally, the nature of poor performance in a CI-fitted population is not yet understood [27]. For example, recent studies [28,29,30] have devoted considerable effort to showing that various factors—including genetic disposition, aetiology, and comorbidities—have an effect on CI outcome. Furthermore, preoperative audiometric assessment in clinical routine [19] potentially provides prognostic value [20,21,22,23]. However, we still see an urgent need for further improvement in preoperative audiometric assessments. Supra-threshold diagnostic tests, such as TDT and other measurements, as earlier applied for topodiagnostics [31,32] in the presence of less sophisticated imaging resources, could experience a renaissance in the preoperative assessment of CI candidates. Retrocochlear lesions can be ruled out by modern imaging, which is superior to audiometry for this purpose [33]. Other retrocochlear deficits can be quantified by the reapplication of established supra-threshold tests and available objective measures [27,34,35]. In the long term, it might be possible to determine a correlation between these additional preoperative diagnostic results and speech recognition with a CI. A clustering according to different patient characteristics may help to solve the enigma of poor performance [27]. If preoperative TD also partially explains the variability in word recognition with CI, then it should be included in future studies. In the past, TDTs were already performed in CI recipients [36,37]. Wable et al. [36] concluded that TD might facilitate further study of the condition of the auditory system in CI recipients as well as help to follow up on possible retrocochlear damage and to link TD to neural survival. Wasman et al. [37] highlighted the potential use of TDTs to explain the spread of outcomes in CI recipients but, because of the small number of study participants, they did not draw clear conclusions. It appears that, together with TD in CI recipients, the preoperative assessment of TD can contribute to a better understanding of the above relationships.

### 4.3. Limits of this Study and Feasibility

The assessment of TD was essentially feasible in our study population. However, the TD assessment took up to 1.5 h. The procedure was perceived as demanding by some of the more elderly study participants. In our experience, some patients may require a more extensive introduction to the test procedure as well as a training run. Furthermore, before the TDT, a precise determination of audiometric thresholds is required; this too can be a challenging task for some patients. Some recipients had difficulties in listening to pure-tone presentations for longer periods. Additionally, tinnitus can be expected to have a detrimental influence on the precision of TDTs. For some patients, the perception of the (pure) test tone may change into a noise-like perception, which can be considered a symptom of possible neural pathologies [14]. Finally, in some patients, two ceiling effects potentially limit the diagnostic value. The first refers to the ceiling of the speech test, while the second may occur in cases where 4FPTA is already poor and the full impact of TD cannot be assessed owing to audiometer limits or the fact that an uncomfortable presentation level has already been reached. To follow up on the results reported here, we plan to continue this study. An increased number of study participants would allow for analysis with respect to even more different patient characteristics, as indicated by the measurement examples in Figure 2.

## 5. Conclusions

The tone decay test is a feasible method for determining tone decay and may contribute to explaining the variability of word recognition scores in hearing aid users with hearing loss in the range 50–80 dB. In cases of disproportionally low aided scores, the tone decay test represents a valuable complement for differential diagnostics. It may provide a better understanding of the limits of hearing aid use in patients considered for cochlear implantation.

## Figures and Tables

**Table 1:** Summary of participants′ demographic and audiometric data.

Subjects	Age	Sex	Ear	Ipsilat. 4FPTA [dB HL]	Contralat. 4FPTA [dB HL]	WRS_max_ [%]	WRS_65_(HA) [%]	Tone Decay at 1, 1.5, 2, 3, 4 kHz [dB]
1	51	f	L	58.8	76.3	0	0	45—n.c.—n.c.—n.c.—n.c.
2	68	m	L	68.8	60.0	55	30	0—n.c.—0—0—n.c.
3	76	m	L	80.0	60.0	90	50	0—10—15—n.c.—n.c.
4	63	m	R	62.5	65.0	65	35	15—n.c.—0—5—10
5	45	m	R	72.5	41.3	75	10	0—0—5—10—30
6	59	m	R	71.3	102.5	0	25	35—10—0—0—25
7	51	f	R	78.8	76.3	45	40	15—n.c.—n.c.—n.c.—n.c.
8	72	f	R	58.8	63.8	75	65	0—5—20—25—20
9	70	f	R	52.5	63.8	90	65	5—5—5—15—15
10	41	m	R	56.3	55.0	65	45	0—n.c.—0—n.c.—5
10	41	m	L	57.5	56.3	85	30	0—n.c.—0—n.c.—0
11	38	f	R	70.0	71.3	80	65	10—0—0—0—10
12	76	f	R	73.8	57.5	15	0	5—15—40—n.c.—20
13	77	m	R	78.8	51.3	75	45	0—0—10—20—10
14	78	f	R	65.0	63.8	65	5	15—n.c.—40—n.c.—45
14	78	f	L	63.8	65.0	45	0	10—n.c.—45—n.c.—55
15	71	f	L	72.5	120	80	75	5—5—5—15—30
16	76	f	R	76.3	63.8	70	0	10—20—25—25—45
16	76	f	L	63.8	76.3	70	15	5—15—30—25—10
17	88	f	R	73.8	38.8	60	35	10—5—5—15—25
18	79	f	R	66.3	62.5	65	10	10—10—20—25—45
18	79	f	L	62.5	66.3	80	25	5—10—20—20—20
19	81	f	R	61.3	57.5	85	60	5—0—0—0—0
19	81	m	L	57.5	61.3	75	45	5—5—5—0—0
20	75	m	L	58.8	52.5	75	70	5—5—5—n.c.—n.c.
21	57	f	L	72.5	83.8	60	40	0—10—10—0—0
22	78	m	L	63.8	41.3	25	35	0—0—5—20—45

n.c.: not conducted.

## Data Availability

Supporting raw data may be obtained through special request from the corresponding autor.
